# The importance of Good Clinical Practice guidelines and its role in clinical trials

**DOI:** 10.2349/biij.4.1.e5

**Published:** 2008-01-01

**Authors:** A Vijayananthan, O Nawawi

**Affiliations:** Department of Biomedical Imaging, Faculty of Medicine, University of Malaya, Kuala Lumpur, Malaysia

**Keywords:** Clinical practice, international, ethical, historical

## Abstract

Good Clinical Practice (GCP) is an international ethical and scientific quality standard for the design, conduct, performance, monitoring, auditing, recording, analyses and reporting of clinical trials. It also serves to protect the rights, integrity and confidentiality of trial subjects. It is very important to understand the background of the formation of the ICH-GCP guidelines as this, in itself, explains the reasons and the need for doing so. In this paper, we address the historical background and the events that led up to the formation of these guidelines. Today, the ICH-GCP guidelines are used in clinical trials throughout the globe with the main aim of protecting and preserving human rights.

## DEFINITION

Good Clinical Practice (GCP) is an international ethical and scientific quality standard for the design, conduct, performance, monitoring, auditing, recording, analyses and reporting of clinical trials. GCP provides assurance that the data and reported results are credible and accurate, and that the rights, integrity and confidentiality of trial subjects are respected and protected [1]. It was finalised in 1996 and became effective in 1997, but was not enforced by law at that time. The Medicines for Human Use (Clinical Trials) Regulations 2004 and the European Union (EU) Directive on Good Clinical Practice changed the world perspective , and compliance with GCP is now a legal obligation in the UK/Europe for all trials involving the investigation of medicinal products [2].

## HISTORICAL BACKGROUND

It is very important to understand the background of the formation of the ICH-GCP guidelines as this, in itself, explains the reasons and the need for doing so ((Table 1)). The concept of the ‘good physician‘ dates back to the ancient world and it is evidenced by the Hippocratic Oath (460 BC). In the United States, the first landmark in the regulation of drugs was the Food and Drugs Act of 1906. This was a result of harmful and lethal drugs that could be bought across the counter just like any other consumer product. Some examples are ‘Grandma’s Secret’ and ‘Kopp’s Baby’s Friend’ which contained large doses of morphine, as well as ‘Dr King’s Consumption Cure‘ and ‘Dr Bull’s Cough Syrup‘ which contained morphine and chloroform [3]. In 1938, the Federal Food, Drug and Cosmetic Act was enacted by the Food and Drug Administration (FDA) and for the first time, manufacturers were required to test drugs for safety and present the evidence of safety testing to the FDA prior to marketing [3].

**Table 1:** Historical background of GCP

460BC	Oath of Hippocrates
1930's	U.S. Food, Drugs and Cosmetic Act
1947	Nuremberg Code
Dec. 10th 1948	Declaration of Human Rights
1962	Kefauver-Harris Amendment
1964, revised 2000	Declaration of Helsinki
1979	The Belmont Report
1982	International Guidelines for Biomedical Research Involving Human Subjects
1996	ICH-GCP guidelines issued
1997	ICH-GCP guidelines becomes law in some countries

In 1947, the Nuremberg Code was created as a result of the unethical and horrific experiments carried out during World War II at Nazi war camps by German physicians, who were subsequently tried and charged at the Nuremberg Military Tribunal. This code states the need for a scientific basis in research on human subjects and voluntary consent and protection of participants [4,5]. The Universal Declaration of Human Rights (December 10th 1948) was also adopted and proclaimed by the United Nations after the atrocities of World War II and it further reiterated the human factor involved in medical experiments.

In 1964, the Declaration of Helsinki was developed by the World Medical Association, forming the basis for the ethical principles that underlie the ICH-GCP guidelines we have today. The focus of this declaration is the protection of the rights of human subjects and this is clear in its introduction [6]:

“The World Medical Association has developed the Declaration of Helsinki as a statement of ethical principles to provide guidance to physicians and other participants in medical research involving human subjects. It is the duty of the physician to promote and safeguard the health of the people. The physician’s knowledge and conscience are dedicated to the fulfilment of this duty”

In 1962 the world was once again shocked by the severe foetal limb deformities linked to the use of maternal thalidomide. In fact this drug reaction was only discovered after 10,000 infants were born in over 20 countries worldwide. In response to this, the Kefauver-Harris Amendments were passed which required the FDA to evaluate all new drugs for safety and efficacy [3].

Another important milestone in the formation of the ICH-GCP guidelines was The Belmont Report which was issued in April 1979 by the National Commission for Protection of Human Subjects of Biomedical and Behavioural Research [7]. The principles of this report are as follows:

1. *Respect for Persons*: This principle acknowledges the dignity and freedom of every person. It requires obtaining informed consent from research subjects (or their legally authorised representatives)
2. *Beneficence*: This principle requires that researchers maximise benefits and minimise harms associated with research. Research-related risks must be reasonable in light of the expected benefits.
3. *Justice*: This principle requires equitable selection and recruitment and fair treatment of research subjects.

In 1982, the World Health Organization (WHO) and the Council for International Organizations of Medical Sciences (CIOMS) issued a document entitled ‘International Guidelines for Biomedical Research Involving Human Subjects‘. This document was released to help developing countries apply the principles of the Declaration of Helsinki and the Nuremberg Code [3]. Worldwide, many organisations and committees issued various documents and guidelines on the same issue, and a decision was taken to consolidate all these guidelines into one universal guideline to be used globally.

In an effort to overcome international GCP inconsistencies throughout the countries, the International Conference for Harmonisation of Technical Requirements for Registration of Pharmaceuticals for Human Use (ICH) issued the ICH Guidelines: Topic E6 Guideline for GCP. This guideline was approved on 17 July 1996 and implemented for clinical trials from 17 January 1997. The participants of these guidelines were representatives of authorities and pharmaceutical companies from the EU, Japan and the United States as well as those of Australia, Canada, the Nordic countries and WHO [8].

## ICH-GCP

The ICH-GCP is a harmonised standard that protects the rights, safety and welfare of human subjects, minimises human exposure to investigational products, improves quality of data, speeds up marketing of new drugs and decreases the cost to sponsors and to the public. Compliance with this standard provides public assurance that the rights, safety and well-being of trial subjects are protected and consistent with the principles of the Declaration of Helsinki, and that the clinical trial data is credible [8]. A historical background of the reasons and the importance of GCP is summarised in (Table 2).

**Table 2:** Reasons for GCP

Increased Ethical Awareness
Improved Trial Methods
Clinical Trial Concept Better Understood
Public/Political Concern over Safety Aspects
Frauds and Accidents during Trials
Growing Research and Development Costs
Increasing Competition
Mutual Recognition of Data
New Market Structure

There are 13 core principles of ICH-GCP and they are as follows:

1. Clinical trials should be conducted in accordance with ethical principles that have their origin in the Declaration of Helsinki, and that are consistent with GCP and the applicable regulatory requirement(s).
2. Before a trial is initiated, foreseeable risks and inconveniences should be weighed against anticipated benefit for the individual trial subject and society. A trial should be initiated and continued only if the anticipated benefits justify the risks.
3. The rights, safety and well-being of the trial subjects are the most important considerations and should prevail over interest of science and society.
4. The available non-clinical and clinical information on an investigational product should be adequate to support the proposed clinical trial.
5. Clinical trials should be scientifically sound, and described in clear, detailed protocol.
6. A trial should be conducted in compliance with the protocol that has received prior institutional review board (IRB)/ independent ethics committee (IEC) approval/favourable opinion.
7. The medical care given to, and medical decisions made on behalf of subjects should always be the responsibility of a qualified physician or, when appropriate, of a qualified dentist.
8. Each individual involved in conducting a trial should be qualified by education, training, and experience to perform his or her respective task(s).
9. Freely given informed consent should be obtained from every subject prior to clinical trial participation.
10. All clinical trial information should be recorded, handled, and stored in a way that allows its accurate reporting, interpretation and verification.
11. The confidentiality of records that could identify subjects should be protected, respecting the privacy and confidentiality rules in accordance with the applicable regulatory requirement(s).
12. Investigational products should be manufactured, handled and stored in accordance with applicable Good Manufacturing Practice (GMP). They should be used in accordance with the approved protocol.
13. Systems with procedures that assure the quality of every aspect of the trial should be implemented.

These principles are self-explanatory and, when summarised, simply mean:

All clinical trials should be conducted in accordance with ethical principles, sound scientific evidence and clear detailed protocols. The benefits of conducting trials should outweigh the risks. The rights, safety and well-being of trial participants are of paramount importance and these should be preserved by obtaining informed consent and maintaining confidentiality. The care must be given by appropriately qualified personnel with adequate experience. Records should be easily accessible and retrievable for accurate reporting, verification and interpretation. Investigational products should be manufactured according to Good Manufacturing Practice (8).

It is also important to mention the participants of GCP in clinical trials and their respective responsibilities. These are summarised in (Table 3).

**Table 3:** GCP participants

Regulatory Authorities	Review submitted clinical data and conduct inspections
The sponsor	Company or institution/organization which takes responsibility for initiation, management and financing of clinical trial
The project monitor	Usually appointed by sponsor
The investigator	Responsible for conduct of clinical trial at the trial site. Team leader.
The pharmacist at trial location	Responsible for maintenance, storage and dispensing of investigational products eg. Drugs in clinical trials
Patients	Human subjects
Ethical review board or Committee for protection of subjects	Appointed by Institution or if not available then the Authoritative Health Body in that Country will be responsible
Committee to monitor large trials	Overseas Sponsors eg. Drug Companies

## GCP IN THE ASIA PACIFIC REGION

Since the conception of the ICH-GCP guidelines, many countries in the Asia-Pacific region realised the need to formulate guidelines of their own based on the framework of the original guidelines [7]. This is clearly seen in (Table 4) that tabulates the adoption of GCP in our country and its neighbours.

**Table 4:** Table 4 GCP Adoption in the Asia Pacific Region

Original ICH-GCP Guidelines	1996
Singapore GCP	1998
Chinese GCP	1999
Malaysian GCP	1999, revised 2004
Thailand	2000
Indonesia	2001

In Malaysia, similar guidelines were formulated in the wake of greater demand by the pharmaceutical industry to conduct clinical trials in the country. The Malaysian Guidelines for GCP was first published in October 1999 and the second edition was released in January 2004. The guideline adopts the basic principle outlined by the International Committee on Harmonization of Good Clinical Practice (ICH-GCP) with some modifications to suit local requirements [1,7].

## CONCLUSION

The importance of GCP lies in the question ‘why’ and ‘how’ GCP trials came about. To know the answer to this, we have to look to the historical background that led to the formulation of GCP guidelines in the United States and Europe and also to the formation of the ICH. The events that led up to the culmination of the ICH-GCP guidelines brought forth public awareness that there was a need to control and regulate clinical trials dealing with drugs and human subjects. The violation of human rights played a large role and that is why the Declaration of Helsinki and The Nuremberg Code remain as the framework of the present guidelines. The ICH-GCP guidelines are therefore considered the ‘bible’ of clinical trials, and have become a global law which safeguards humanity as we know it today.
